# Phylogeny of minute carabid beetles and their relatives based upon DNA sequence data (Coleoptera, Carabidae, Trechitae)

**DOI:** 10.3897/zookeys.147.1871

**Published:** 2011-11-16

**Authors:** David R. Maddison, Karen A. Ober

**Affiliations:** 1 Department of Entomology, University of Arizona, Tucson, AZ 85721, USA; Current address: Department of Zoology, 3029 Cordley Hall, Oregon State University, Corvallis, OR 97331, USA; 2 Department of Entomology, University of Arizona, Tucson, AZ 85721, USA; Current Address: Department of Biology, College of the Holy Cross, Worcester, MA 01610, USA

**Keywords:** ground beetles, DNA, molecular phylogeny, Bembidiini, Trechinae, Carabidae, Trechitae

## Abstract

The phylogeny of ground beetles of supertribe Trechitae is inferred using DNA sequences of genes that code for 28S ribosomal RNA, 18S ribosomal RNA, and *wingless*. Within the outgroups, austral psydrines are inferred to be monophyletic, and separate from the three genera of true Psydrina (*Psydrus*, *Nomius*, *Laccocenus*); the austral psydrines are formally removed from Psydrini and are treated herein as their own tribe, Moriomorphini Sloane. All three genes place *Gehringia* with Psydrina. Trechitae is inferred to be monophyletic, and sister to Patrobini.

Within trechites, evidence is presented that *Tasmanitachoides* is not a tachyine, but is instead a member of Trechini. *Perileptus* is a member of subtribe Trechodina. Against Erwin’s hypothesis of anillines as a polyphyletic lineage derived from the tachyine genus *Paratachys*, the anillines sampled are monophyletic, and not related to *Paratachys*. Zolini, Pogonini, Tachyina, and Xystosomina are all monophyletic, with the latter two being sister groups. The relationships of the subtribe Bembidiina were studied in greater detail. *Phrypeus* is only distantly related to *Bembidion*, and there is no evidence from sequence data that it belongs within Bembidiina. Three groups that have been recently considered to be outside of the large genus *Bembidion* are shown to be derived members of *Bembidion*, related to subgroups: *Cillenus* is related to the *Ocydromus* complex of *Bembidion*, *Zecillenus* is related to the New Zealand subgenus *Zeplataphus*, and *Hydrium* is close to subgenus *Metallina*. The relationships among major lineages of Trechitae are not, however, resolved with these data.

## Introduction

The supertribe Trechitae comprises over 5,300 described species (Lorenz 2005) of ground beetles. Although this is comparable to the number of mammal species (Wilson and Reeder 2005), trechites are much more poorly known. Trechites are diverse on all continents except Antarctica. Most adults of this group are relatively small (less than 10 mm in length), and include the smallest known carabids, about 0.7 mm in length (Erwin 1973; Jeannel 1963). Division of this group into suprageneric taxa varies among authors, with most North American authors favoring four tribes: Trechini and Bembidiini, with over 2,500 species each, and the smaller groups Pogonini and Zolini, with about 85 and 55 species respectively (Lorenz 2005). Trechini includes many troglobitic species, and is most speciose in temperate areas. Bembidiini is worldwide, with many species living along bodies of water; it includes the smallest adults. This tribe includes the largest carabid genus, *Bembidion*, with over 1,200 recognized species (Lorenz 2005). Most pogonines are halobiontic; the majority live in the Old World. The Zolini is a strictly south-temperate lineage, except for the monotypic genus *Sinozolus* from China (Deuve 1997). A brief review of the diversity within each tribe is given in Grebennikov and Maddison (2005).

**Figure 1.**
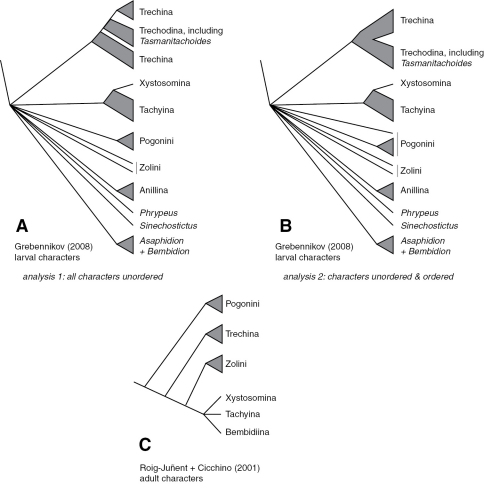
Phylogenies of Trechitae from morphological studies **A** Strict consensus tree of most parsimonious trees from larval data, with all characters treated as unordered, from Grebennikov (2008); this is the tree presented in Grebennikov (2008: Fig. 3) **B** Strict consensus tree of most parsimonious trees from larval data, with some characters treated as ordered, as specified by Grebennikov (2008); this tree is not presented in that paper, but was inferred from the described conditions **C** “Best fit” tree presented by Roig-Juñent and Cicchino (2001) based upon adult morphological data.

The basic structure of the phylogeny of trechites is not well known. The only explicit, modern analyses have been based upon limited characters of adult and larval structure (Grebennikov 2008; Grebennikov and Maddison 2005; Roig-Juñent and Cicchino 2001); these have inferred a few aspects of the phylogeny (Fig. 1).

We present here the first detailed examination of relationships within Trechitae based upon DNA sequences, using portions of genes for small (18S) and large (28S) subunits of ribosomal RNA, as well as the nuclear protein-coding gene *wingless*.

This paper has been over a decade in gestation, and some results have already been reported in other publications. For example, the discovery from the DNA sequence data reported herein of *Tasmanitachoides* place in Trechini rather than in Tachyina was the inspiration for Grebennikov’s search for *Tasmanitachoides* larvae, which as he recently reported (Grebennikov 2008) confirms its placement in Trechini.

## Methods

**Taxa examined**. The 14 outgroup species included are listed in Table 1. Morphological data and previously collected 18S rDNA data suggests that the sister group of Trechitae is likely Patrobini (Arndt 1993; Deuve 1993; Maddison et al. 1999a; Müller 1975; Zamotajlov 2002), and we include three species of this near outgroup. More distant relatives are less clear. We include representatives of carabid groups that are of a similar grade as trechites, that is, they are members of Carabidae Conjunctae but are not members of Harpalinae or Brachininae. These include all three genera of Psydrini (s.str.), as well as six genera of Moriomorphini. The latter is the group referred to as “austral psydrines” in Maddison et al. (1999a), and includes all traditional psydrines except for Psydrini in the strict sense. In addition, as Gehringiini has been proposed to be a psydrine relative (Erwin 1985), or potentially within Trechitae (Erwin 1984), we include one member of Gehringiini, *Gehringia olympica* Darlington. We also include one representative of Harpalinae, *Pterostichus*, as the most-distant outgroup.

**Table 1.** Outgroup taxon sampling. Four-digit numbers in entries are D.R. Maddison voucher numbers; further information on the specimens is given in the Appendix; where two numbers are listed, the sequence was formed by combining data from both specimens. Other entries are GenBank numbers of previously published sequences from Maddison et al. (1999a, 1999b), Ober (2002), and Ober and Maddison (2008).

	**18S**	**28S**	***swingles***
**Pterostichini**			
*Pterostichus melanarius* Illiger	AF002779	AF398707	AF398623
**Moriomorphini**			
*Amblytelus curtus* (Fabricius)	AF012484	AF398683	AF398566
*Mecyclothorax vulcanus* (Blackburn)	AF012482	AF398648	AF398601
*Melisodera picipennis* Westwood	AF012481	AF398640	AF398602
*Meonis* sp.	AF398722	AF398692	AF398603
*Sitaphe parallelipennis* Baehr	0669	2247	0669
*Tropopterus* sp.	AF012483	2200	2200
**Psydrini**			
*Laccocenus ambiguus* Sloane	AF012486	AF398675	AF398596
*Psydrus piceus* LeConte	AF002784	AF398684	1627
*Nomius pygmaeus* (Dejean)	0893	AF438100	AF437971
**Gehringiini**			
*Gehringia olympica* Darlington	AF012512	AF398702	AF398591
**Patrobini**			
*Diplous californicus* (Motschulsky)	AF002785	AF398699	AF398587
*Patrobus longicornis* (Say)	AF002786	AF398700	AF398613
*Penetretus temporalis* Bedel	0631,1710	0631	0631

Within Trechitae, 64 species in 40 genera are sampled, with all tribes represented, and an emphasis on subtribe Bembidiina (Table 2). The classification used here is modified version of Lorenz’s (2005), with ranks similar to those typically used in North America (e.g., Lindroth 1963). The sequences obtained for *Trechus* came from two different specimens from Montana; one of these is *Trechus oregonensis* Hatch, the other, a female, cannot be identified with certainty to species, but belongs to the *Trechus chalybeus* species group, to which *Trechus oregonensis* also belongs. In analyses combining different genes, sequences from these two individuals were combined into a chimeric taxon.

**Table 2.** Taxon sampling of trechites. Four-digit numbers in entries are D.R. Maddison voucher numbers; further information on the specimens is given in the Appendix; where two numbers are listed, the sequence was formed by combining data from both specimens. Other entries are GenBank numbers of previously published sequences from Maddison et al. (1999a, 1999b), Ober (2002), and Ober and Maddison (2008).

	**18S**	**28S**	**s*wingles***
**Trechini: Trechodina**			
*Cnides* sp.	1808	0691	
*Pachydesus* sp.	0678	AF438112	AF437978
*Perileptus areolatus* (Creutzer)	1707	0824	1707
*Perileptus sloanei* Moore		0767	
*Thalassophilus longicornis* (Sturm)		0823	0823
*Trechodes bipartitus* (MacLeay)	0705	0705	0705
*Trechodes jeanneli jeanneli* Mateu		0606	
*Trechosiella* sp.	1709	0723	1709
*Tasmanitachoides fitzroyi* (Darlington)	1575	0762	
**Trechini: Trechina**			
*Trechus chalybeus* species group	AF002793		
*Trechus oregonensis* Hatch		AF398673	0587
*Omalodera limbata* Blanchard	0571	0571	0571
*Homaloderodes germaini* Jeannel		1066	
*Kenodactylus audouini* (Guérin-Méneville)		0670	0670
*Paratrechus* sp.		1076	
*Trechinotus flavocinctus* Jeannel	0575	0575	0575
**Zolini**			
*Merizodus angusticollis* Solier	AF012487	0453	0453
*Oopterus* sp.	AF012488	0387	0387
*Oopterus helmsi* (Sharp)	AF002787	0354	0354
*Sloaneana tasmaniae* (Sloane)	AF002788	0339	0339
**Pogonini**			
*Diplochaetus planatus* (G.H. Horn)	AF002789	AF438060	AF437938
*Pogonus (Pogonus) chalceus* (Marsham)	1711	0679	0679
*Thalassotrechus barbarae* (G.H.Horn)		0703	0530
**Bembidiini: Tachyina**			
*Lymnastis* sp.	0988	0988	0988
*Micratopus* sp.		0605	0605
*Paratachys vorax* (LeConte)		0410	0410
*Elaphropus obesulus* LeConte	0411	0411	0411
*Elaphropus* cf. *nigrolimbatus* Peringuey		0761	
*Elaphropus* sp. 3		0713	0713
*Pericompsus laetulus* LeConte	AF002790	0429	0429
*Polyderis rufotestacea* (Hayward)		0717, 0718	0718
*Tachys vittiger* LeConte		0760	
*Tachys corax* LeConte		0604	0604
*Tachyta nana inornata* (Say)	0573	AF438141	AF438002
**Bembidiini: Xystosomina**			
*Erwiniana hilaris* (Bates)	AF012489	0409	0409
*Erwiniana crassa* (Erwin)		0989	0989
*Mioptachys flavicauda* Say	0684	0684	0684
*Philipis bicolor* Baehr		0592	0592
**Bembidiini: Anillina**			
*Anillinus* (*langdoni* group) sp.		0690	0690
*Serranillus* sp.	1084	1084	
*Typhlocharis armata* Coiffait	0572,1718	0572	0572
*Nesamblyops* sp.		0696	
**Bembidiini: Bembidiina**			
*Asaphidion alaskanum* Wickham		0585	0585
*Asaphidion championi* Andrewes		0574	
*Asaphidion curtum* (Heyden)	AF002792	0267	0267
*Amerizus* (*Amerizus*) sp.		0576	0576
*Ocys harpaloides* (Audinet-Serville)		0569	0569
*Phrypeus rickseckeri* Hayward		0776	0692
*Sinechostichus solarii* (G. Müller)		0603	0603
*Bembidion* (*Antiperyphanes*)sp. nr. *chilense* Solier		0714	0714
*Bembidion* (*Hoquedela*) *cf. csikii* Jedlicka		0916	0916
*Bembidion (Cillenus) laterale* (Samouelle)		0602	0602
*Bembidion (Notaphus) insulatum* (LeConte)		0444	0444
*Bembidion (Bracteon) balli* Lindroth	EF648613	EF648838	EF649474
*Bembidion (Odontium) coxendix* Say	EF648618	EF648837	EF649481
*Bembidion (Metallina) dyschirinum* LeConte		0896	0896
*Bembidion (Pseudoperyphus) integrum* Casey	EF648659	EF649056	EF649609
*Bembidion (Bracteon) levettei carrianum* Casey	EF648620	EF648842	EF649480
*Bembidion (Hydrium) levigatum* Say		0763	0763
*Bembidion (Ocydromus) mexicanum* Dejean	AF012490	0260	0262
*Bembidion (Phyla) obtusum* Audinet-Serville		0895	0895
*Bembidion (Melomalus) planatum* (LeConte)		0601	0601
*Bembidion (Bembidion) quadrimaculatum dubitans* (LeConte)	0676	0676	0676
*Bembidion (Zeplataphus) tairuense* Bates	0607	0607	0607
*Bembidion (Zecillenus)* sp.	0595	0595	0595

Locality information for the specimens newly sequenced in this paper is given in the Appendix. Voucher specimens are deposited in the David Maddison voucher collection in the Oregon State Arthropod Collection at Oregon State University.

**DNA sequencing**. Methods for obtaining DNA sequences, including extraction methods and cycling reactions, are described in Maddison et al. (2008). Primers used are listed in Table 3; see Maddison et al. (2008) for information about original source of primer sequences. In brief, we obtained *ca.* 2000 bases of sequence data of 18S ribosomal DNA (18S rDNA or 18S), about 1000 bases in the D1 through D3 domains of 28S ribosomal DNA (28S rDNA, or 28S) and about 450 bases of the nuclear protein-coding gene *wingless* (*wg*). Amplified products were cleaned, quantified, and sequenced at the University of Arizona’s Genomic and Technology Core Facility using either a 3730 or 3730 XL Applied Biosystems automatic sequencer.

**Table 3.** Primers used for DNA amplification and sequencing. Dir: direction of primer, either forward (F) or reverse (R). Syn: primer synonym. Kind: primer used for original PCR amplification and sequencing (A) or primer used only for sequencing (S). Original references for primer sequences are given in Maddison et al. (2008). Primer pairs used in earlier PCR reactions for *wingless* were 5wg-3wg, 5wgB-3wg2, and B5wg1-B3wg2; more recent, and reliable, reactions used the pairs wg550F-wgABRz or wg578F-wgABR.

Gene	Primer	Syn	Dir	Kind	Sequence
28S	LS58F	D1	F	A	GGGAGGAAAAGAAACTAAC
	LS998R	D3	R	A	GCATAGTTCACCATCTTTC
18S	SS27F	518S	F	A	TATGCTTGTCTCAAAGATTAA
	S1893R	18L	R	A	CACCYACGGAAACCTTGTTACGACTT
	SS398F	18Sai	F	S	CCTGAGAAACGGCTACCACATC
	SS1054F	760F	F	S	ATCAAGAACGAAAGT
	SS1090R	18Sbi	R	S	GAGTCTCGTTCGTTATCGGA
	SS1554R	909R	R	S	GTCCTGTTCCATTATTCCAT
wg	wg550F		F	A	ATGCGTCAGGARTGYAARTGYCAYGGYATGTC
	wgAbRZ		R	A	CACTTNACYTCRCARCACCARTG
	wg578F		F	A	TGCACNGTGAARACYTGCTGGATG
	wgAbR		R	A	YTCGCAGCACCARTGGAA
	B5wg1		F	A	GARTGYAAGTGTCAYGGYATGTCTGG
	5wg		F	A	GARTGYAARTCYCAYGGYATGTCTGG
	5wgB		F	A	ACBTGYTGGATGCGNCTKCC
	3wg2		R	A	CTCGCARCACCARTGGAATGTRCA
	B3wg2		R	A	ACTCGCARCACCAGTGGAATGTRCA
	3wg		R	A	ACTCGCARCACCARTGGAATGTRCA

Assembly of multiple chromatograms for each gene fragment and initial base calls were made with Sequencher (Gene Codes Corporation) or using Phred (Green and Ewing 2002) and Phrap (Green 1999) as orchestrated by Mesquite’s Chromaseq package (Maddison and Maddison 2009a; Maddison and Maddison 2009b), with subsequent modifications by Chromaseq and manual inspection. Multiple peaks at a single position in both reads were coded using IUPAC ambiguity codes.

Newly obtained sequences have been deposited in GenBank with accession numbers GU556024 through GU556153.

**Alignment and resulting matrices**. The two ribosomal genes, 18S and 28S, were aligned using ClustalW 1.8.3, with a gap opening cost of 10, gap extension of 0.1, then adjusted by eye; areas of uncertain alignment were excluded.

The amino acid translation of the *wingless* gene was aligned using Clustal W version 1.83 (Chenna et al. 2003) using gap opening cost of 5, gap extension cost 0.2, and a Gonnet series matrix. The central region of the *wingless* alignment evidently had a rich history of insertion and deletions; the alignment of this region was adjusted by eye in MacClade (Maddison and Maddison 2005). An alignment of nucleotides was then created, with the nucleotides forced to match the amino acid alignment using MacClade. There were two *wingless* matrices produced, one with the alignment-ambiguous region included (“all nucleotides”), and another with that region excluded (“well-aligned nucleotides”).

**Phylogenetic inference.** Each of the four matrices (28S, 18S, and the two *wingless* matrices) were subjected to parsimony, Bayesian, and maximum likelihood analyses.

Most-parsimonious trees were sought using PAUP* (Swofford 2002). For each search, 2000 replicates were conducted, each beginning with a starting tree formed by the random addition sequence option, with subsequent TBR branch rearrangement. The number of most parsimonious trees (MPTs) ranged from 15 to 502 across the four matrices, and for each matrix the MPTs were found in at least 460 of the 2000 replicates.

For parsimony bootstrap analyses in PAUP*, 1000 bootstrap replicates were conducted, each of which used a heuristic search with five replicates, each beginning with a starting tree formed by the random addition sequence option, with TBR branch rearrangement, with each replicate saving no more than 25 trees.

Models of nucleotide evolution chosen with the aid of ModelTest (Posada 2005), with the aid of PAUP* (Swofford 2002). For 18S and 28S genes, the model chosen by the Akaike Information Criterion (AIC) was a General Time Reversible rate matrix with a proportion of sites being invariant and the remainder following a gamma distribution (the GTR+I+Γ model). For the *wingless* gene, the GTR+I+Γ model was chosen for the region without extensive insertions and deletions, but for the indel-rich region a GTR+Γ model was preferred. When codon positions were allowed separate models, GTR+I+Γ was preferred for first positions, GTR+Γ for second positions, and HKY85+I+Γ for third positions.

Bayesian analyses were conducted using MrBayes (Huelsenbeck and Ronquist 2005). Two runs of four chains each were run for between 8 million and 30 million generations, with trees sampled every 1,000 generations. Runs were terminated once the average standard deviation of split frequencies went below 0.01 (Huelsenbeck and Ronquist 2005). For each analysis, the trees in a burn-in period were excluded, and the majority-rule consensus tree of remaining trees was calculated by PAUP to determine Bayesian Posterior Probabilities (BPP) of clades. The burn-in period was at least 25% of the total length of the run (as only the remaining 75% were used to calculate the average standard deviation of split frequencies used as a convergence diagnostic), and extended until the likelihood scores and all parameter values reached a stable plateau, as judged by visualization tools in Tracer (Rambaut and Drummond 2004). The burn-in period ranged from 3 million generations to 25 million generations. The number of trees sampled for each analysis varied from 10,000 to 30,000.

Likelihood analyses of nucleotide data were conducted using RAxML version 7.0.4 (Stamatakis 2006). For each matrix, 1000 search replicates were conducted to find the maximum likelihood trees. 2000 non-parametric bootstrap replicates were used to calculate bootstrap values for groups of interest.

Several of the analyses of 18S rDNA yielded trees with the trechine *Cnides*, whose terminal branch was extremely long, well outside of Trechitae. As morphological data indicates definitively that *Cnides* is a trechite, some analyses were performed that forced it to reside within Trechitae. Two full suites of constrained analyses were conducted, one with Trechitae constrained to be monophyletic, and other with Trechini constrained to be monophyletic. For the latter, the position of *Tasmanitachoides* was not constrained in likelihood and parsimony analyses, allowing it to move anywhere on the tree.

All trees are shown rooted within the outgroup, arbitrarily next to *Pterostichus*.

Grebennikov’s (2008) larval morphological data were reanalyzed using TNT version 1.1 (Goloboff et al. 2008). Most parsimonious trees were found using the following commands: rseed[, hold 1000, xmult: hits 100 ratchet 5 norss nocss, xmult. This caused TNT to do multiple searches, each beginning with a tree with taxa added in random order, with up to 1000 trees held in memory, with each search using five cycles of ratcheting; enough searches were done until the best trees found were found 100 times. 332 equally parsimonious trees of length 140 were found.

## Results of phylogenetic analysis

**Figure 2.**
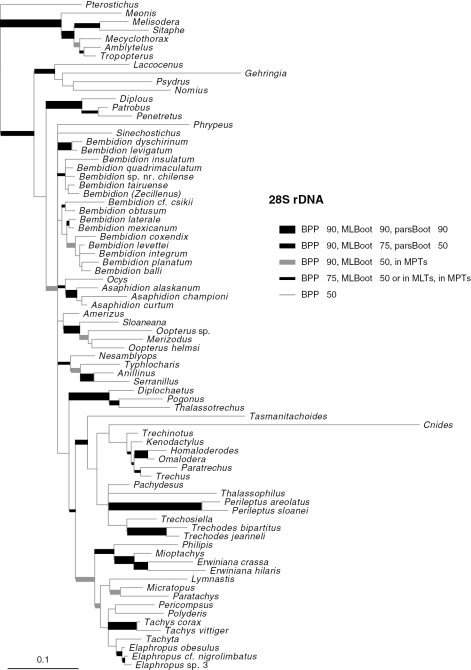
Majority-rule consensus tree of trees sampled in Bayesian analysis, with branch lengths proportional to average branch lengths across trees that contain that branch, for 28S rDNA data. Branch lengths were reconstructed by MrBayes; scale bar units are substitutions per site. Thickness and shade of branches indicate support for that clade, based upon estimated Bayesian Posterior Probability percentages (BPP), Maximum Likelihood bootstrap values (MLBoot), and parsimony bootstrap values (parsBoot).

**Figure 3.**
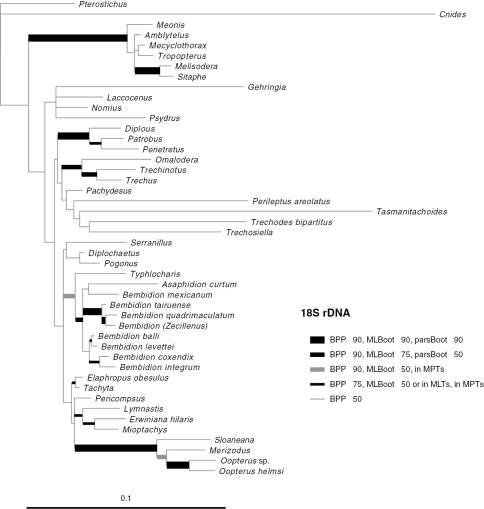
Majority-rule consensus tree of trees sampled in Bayesian analysis, with branch lengths proportional to average branch lengths across trees that contain that branch, for 18S rDNA data. See caption of Fig. 2 for additional details.

**Figure 4.**
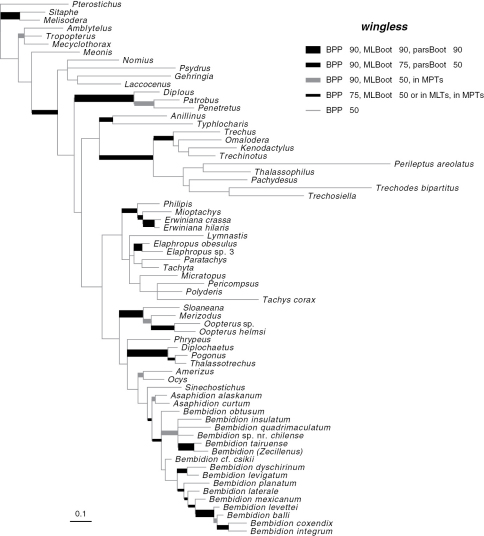
Majority-rule consensus tree of trees sampled in Bayesian analysis, with branch lengths proportional to average branch lengths across trees that contain that branch, for the complete *wingless* data. See caption of Fig. 2 for additional details.

**Figure 5.**
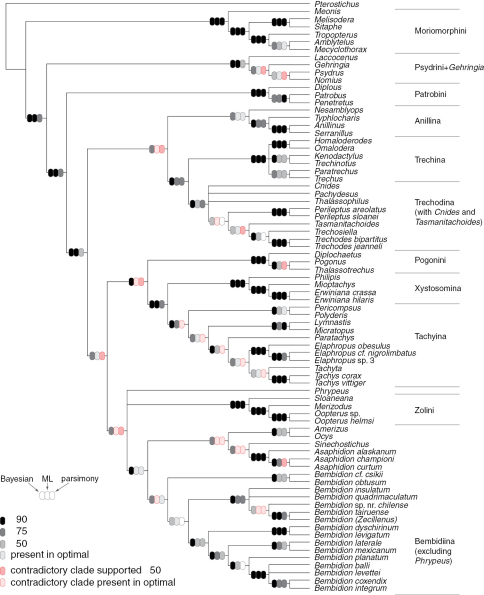
Majority-rule consensus tree of trees sampled in Bayesian analysis for all three genes analyzed together. Ovals on branches indicate support for the clade based upon Bayesian (left), maximum likelihood (center), and parsimony (right) analyses. Darkest tones indicate strongest support for (grays and black) or against (pinks) the clade, with values indicating posterior probability expressed as a percentage (Bayesian), or bootstrap percentage (likelihood and parsimony).

**Figure 6.**
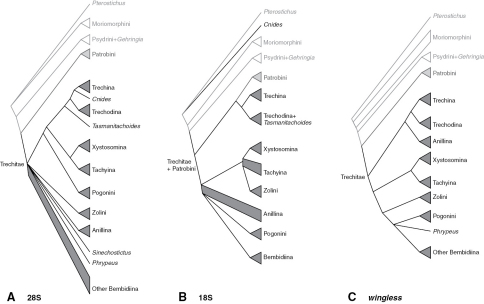
Summary of subtribal and tribal relationships supported by individual genes. Triangles indicate monophyletic groups; quadrangles represent paraphyletic groups **A** 28S rDNA **B** 18S rDNA **C** *wingless*.

**Figure 7.**
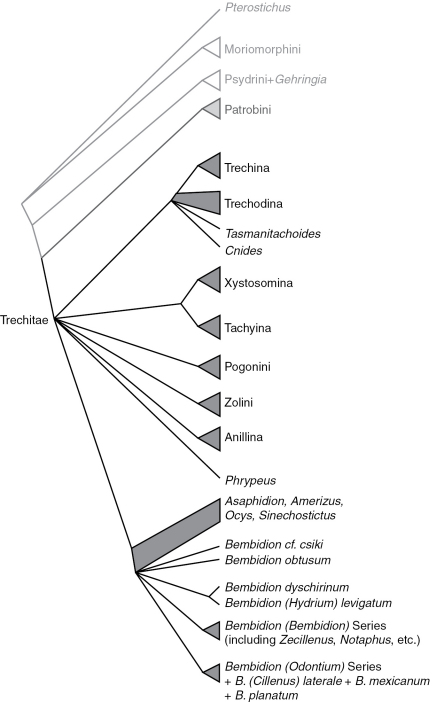
Summary of relationships in Trechitae and related taxa. Branches (including those subtended by triangles) indicate monophyletic groups supported by the combined analyses and at least two of the genes; quadrangles indicate groups whose status is unresolved.

Phylogenetic trees inferred from individual genes are shown in Figs 2–4, and from the merged matrix in Fig. 5. Support values for various hypotheses are shown in Tables 4 and 5. Summaries of supported phylogenetic hypotheses are presented in Figs 6, 7.

**Table 4.** Support values for various groups outside of Bembidiini (*sensu lat.*): B: Bayesian posterior probability, expressed as a percentage; ML: Maximum likelihood analysis; P: parsimony analysis. For maximum likelihood and parsimony analyses, numbers indicate the bootstrap support expressed as a percentage; check marks indicate that the clade is present in the optimal (maximum likelihood or post parsimonious) trees but with bootstrap value below 50; x indicates that a contradictory clade was present in the optimal (maximum likelihood or post parsimonious) trees but with bootstrap value below 50; negative values indicate Bayesian posterior probability or bootstrap support for a contradictory clade. Boxes in gray to black indicate support for the clade; boxes in pink to red indicate support against that clade, with darker colors indicating stronger support. Dashes indicate no support for or against the clade because of insufficient taxon sampling for that gene; blank boxes indicate no support for or against the clade because of lack of resolution in the inferred trees. Abbreviations: “inc.” = “including”, “exc.” = “excluding”.

	**merged**			**28S rDNA**			**18S rDNA**			**wg, all nucleotides**			**wg, well-aligned nucleotides**		
	B	ML	P	B	ML	P	B	ML	P	B	ML	P	B	ML	P
Moriomorphini	100	100	100	100	100	100	100	100	100	-100	-78		-75	x	✓
Psydrini+ *Gehringia*	100	99	70	100	99	70	56	x	x	88	✓		88	✓	✓
Patrobini+ Trechitae	100	92	80	54	58	66	-66	x	x	100	58		100	59	✓
Trechitae	100	93	66	100	70	53	-66	x	x	100	77		100	75	✓
Trechini	99	79	86	100	83	64	-66	x	x	100	98	78	100	98	75
Trechina	100	95	98	62	70	96	100	80	70	100	99	80	100	100	79
Trechodina	94	64	78	-66	x	✓	-89	x	x	100	82		100	79	
*Tasman*. with Trechini	99	79	86	100	83	64	95	✓	60	-	-	-	-	-	-
Pogonini	100	100	100	100	98	97	100	72	72	100	100	100	100	100	100
Zolini	100	100	100	100	96	96	100	100	100	100	99	97	100	97	92

**Table 5.** Support values for various groups of Bembidiini *(sens. lat.)*. See legend of Table 4 for more explanation.

	**merged**			**28S rDNA**			**18S rDNA**			**wg, all nucleotides**			**wg, well-aligned nucleotides**		
	B	ML	P	B	ML	P	B	ML	P	B	ML	P	B	ML	P
Bembidiini (sens. lat.)	-88	x	x	-85	x	x	-98	x	x	-100	-63	x	-100	-56	x
Tachyina+ Xystosomina	100	92	85	100	72	66	-62	x	x	68	x	x	80	✓	✓
Tachyina	100	88	x	99	67	x	-62	x	x	99	✓	x	87	✓	x
Xystosomina	100	99	100	100	80	75	89	71	69	99	88	85	100	88	89
Anillina (inc. *Nesamblyops*)	87	✓	✓	93	✓	✓	-	-	-	-	-	-	-	-	-
Bembidiina inc. *Phrypeus*		x	x		x	x	-	-	-	-	x		51	x	x
Bembidiina exc. *Phrypeus*	91	✓	✓		x	x	100	76	78	100	✓	✓	89	x	x
*Bembidion* (sens. lat.)	75	x	✓		x		-54	x	-57	89	✓	✓	83	x	
*Zecillenus* in *Bembidion*	100	91	89	91	✓	✓	100	98	98	100	98	96	100	95	93
*Bembidion taiurense* + *Zecillenus*	100	91	89	52	✓	✓	-100	-91	-91	100	98	96	100	95	93
*Bembidion* series	100	87	88	91	✓	✓	100	98	98	98	60	67	97	57	66
*Cillenus* in *Bembidion*	100	84	64	99	79	78	-	-	-	99	✓	✓	93	✓	✓

Support values for various groups in analyses of 18S constrained to keep Trechitae or Trechini monophyletic are shown in Table 6.

Results for the reanalysis of Grebennikov’s (2008) larval data are presented in Fig. 1B, and described in more detail in the text, below.

**Table 6.** Support values for various groups outside of Bembidiini (*sensu lat.*): comparison of results for 18S rDNA between unconstrained and constrained analyses. For the “Trechitae constrained” analyses, all of Trechitae was constrained to be monophyletic. For the “Trechini constrained analyses”, all of Trechini was constrained to be monophyletic for the Bayesian analysis; for the remaining analyses, Trechini was constrained to be monophyletic, except that *Tasmanitachoides* was unconstrained, and could move anywhere in the tree if that were optimal. “n/a” indicates that support values for that group are irrelevant as the group was forced to be monophyletic; for meaning of other symbols and colors, see legend of Table 4.

	**No Constraints**			**Trechitae constrained**			**Trechini constrained**		
	B	ML	P	B	ML	P	B	ML	P
Psydrini+ *Gehringia*	56	x	x	59	x	-63	50	✓	x
Patrobini+ Trechitae	-66	x	x	99	95	94	94	✓	53
Trechitae	-66	x	x	n/a	n/a	n/a		x	✓
Trechini	-66	x	x	-85	x	✓	n/a	n/a	n/a
Trechina	100	80	70	99	82	69	99	82	70
Trechodina	-89	x	x	-85	x		64	3	x
*Tasman*. with Trechini	95	✓	60	89	✓	67	100	76	84

## Discussion

We here discuss in turn the evidence available for various relationships within trechites. As significant results have been found within the outgroups sampled, we will discuss these first.

### Outgroup structure: Monophyly of Moriomorphini

Among the carabids currently considered to belong to Psydrini (*sens. lat*.), only three genera belong to Psydrina in the strict sense: *Psydrus* (North America), *Nomius* (Holarctic and Africa), and *Laccocenus* (Australia). The remaining genera are arrayed in multiple subtribes (Baehr 1998; Moore 1963), and are restricted to the Southern Hemisphere, primarily in temperate areas, except for members of the genus *Mecyclothorax*, which occur as far north as Hawai’i (Liebherr 2006; 2008; 2009). Sequences of 18S rDNA indicate that all psydrines other than *Psydrus*, *Nomius*, and *Laccocenus* belong to a clade, termed the “austral psydrines” by Maddison et al. (1999a). Three apomorphies of adult structure (Baehr 1999) also indicate monophyly of the austral psydrines. In addition, 18S rDNA indicated that austral psydrines were not closely related to psydrines in the strict sense (Maddison, et al. 1999a).

Our data indicate that Psydrina are not closely related to austral psydrines. While a strong test of this hypothesis with 28S and *wingless* would require more extensive sampling of non-trechites than we have done, all three genes we studied suggest that Psydrini in the classical sense, containing *Psydrus*, *Nomius*, *Laccocenus*, and the austral psydrines, is not monophyletic (Figs 2–5). Combined with evidence provided by the more extensive 18S rDNA taxon sampling of Maddison *et al.* (1999a), and the morphological data (Baehr 1999), these two groups should be in separate taxa. We therefore remove austral psydrines from Psydrini, and place them in their own tribe. The valid name for this tribe is Moriomorphini Sloane (1890: 646).

Our results indicate strong support for monophyly of Moriomorphini from both 18S rDNA and 28S rDNA (Table 4). Most analyses of the *wingless* gene suggest instead that the moriomorphines form a grade, although parsimony analysis of the well-aligned nucleotides does support monophyly of the group. More extensive sampling of *wingless* sequences of non-trechites is needed to examine this further.

### Outgroup structure: relationship of Gehringiini

Gehringiini are a small group (five known species; Baehr et al. 2009) of minute carabids of uncertain relationships. Morphological data places them variously as sister group of Paussinae (R.T. Bell at the 1983 Entomological Society of America meetings; Beutel 1992), with Trachypachidae (Kryzhanovskij 1976; Lindroth 1969), among the basal carabids (Bell 1967; Darlington 1933; Jeannel 1941), or a member of Trechitae or a relative of Psydrini (Bell 1967; Darlington 1933; Erwin 1984; 1985; Hammond 1979). Sequences of 18S rDNA suggested a possible placement near *Cymbionotum* (Maddison et al. 1999a). As most of the groups that have been proposed as near-relatives of gehringiines are not included in the current study, we cannot conduct a definitive test. However, it is remarkable that separate analyses of each of the three genes, plus the combined analyses, indicate at least some level of support for having *Gehringia* belonging to a clade with the true Psydrini (Figs 2–5, Table 4), echoing the placement “as a basal psydrite group” by Erwin (1985).

### Relationships of Patrobini to Trechitae

Trechitae + Patrobini share a number of synapomorphies in adult structure (Zamotajlov 2002), larval characteristics (Arndt 1993; Arndt 1998; Bousquet and Grebennikov 1999; Houston and Luff 1975; Müller 1975; Zamotajlov 2002), and female abdominal structure (Deuve 1993) that suggest they form a clade. This result was corroborated by 18S rDNA (Maddison et al. 1999a). In contrast, Roig-Juñent and Cicchino (2001) has the morimorphine subgroup Amblytelini as sister to Patrobini, which are together sister to Trechitae.

Consistent with most morphological data, our 28S and *wingless* data support monophyly of Patrobini plus Trechitae (Table 5), as does 18S if Trechini is constrained to be monophyletic (Table 6).

### Monophyly of Trechitae

Shared derived characters that provide evidence for monophyly of Trechitae are found in multiple character systems. Protarsomeres of adult males are uniquely dentate and dilated on the mesal side (Roig-Juñent and Cicchino 2001). There are derived traits of larval structure (Arndt 1993; Grebennikov and Maddison 2000; 2005). Males lack chiasmata in meiosis (Serrano 1981), although this has been examined in relatively few genera, and in no zolines. Males of almost all other carabids, including patrobines, are chiasmatic (Galián et al. 1994; Serrano and Galian 1998), with the possible exception of isolated separate origins within the distantly-related Carabini (Yadav and Burra 1987) and Harpalini (Serrano 1981). The lack of chiasmata in trechites is thus a notable synapomorphy. 18S rDNA has also provided evidence of monophyly (Maddison, et al. 1999a).

Trechitae is strongly supported as monophyletic in 28S, *wingless*, and the merged matrix (Table 5). In contrast, because of the placement of *Cnides* outside of Trechitae (Fig. 3), as discussed in the next section, 18S provides evidence against the monophyly of Trechitae. If *Cnides* is forced to stay within Trechini, however, 18S provides no clear signal for or against monophyly (Table 6).

### Monophyly and phylogeny of Trechini

The primary synapomorphy suggesting the monophyly of Trechini is the presence of deep furrows on the dorsal surface of the head (Jeannel 1926). In its extreme form, this state is unique within carabids, but there are also trechines with relatively shallow, less extensive furrows that are not dissimilar to those found in other carabids. There are, however, several derived states in larval characters that indicate monophyly of Trechini (Grebennikov 2008; Grebennikov and Maddison 2005).

Our results from 28S, *wingless*, and the merged matrices indicate strong support for monophyly of Trechini (Table 4), if *Tasmanitachoides* is included within the tribe (as discussed in the next section). In contrast, 18S provides moderate evidence against the monophyly of trechines, because of the placement of *Cnides* outside of Trechitae (Fig. 3). However, the exceedingly divergent *Cnides* 18S sequence (note the length of its branch in Fig. 3) makes artificial attraction of the long branch (Felsenstein 1978) to distantly related outgroups a reasonable explanation.

In Jeannel’s great work (1926; 1927; 1928; 1930) trechines are divided into five groups. One of most distinctive groups are the trechodines, a predominately Southern-Hemisphere group, characterized by a basal bulb of the male aedeagus that is open dorsally, as opposed to the closed basal bulb found in other trechines. Among the taxa we have sequenced, trechodines are represented by *Pachydesus*, *Thalassophilus*, *Cnides*, and *Trechodes*. Jeannel’s other four groups are each represented in our data: his aepines by *Kenodactylus*, his homaloderines by *Omalodera*, *Homaloderodes*, and *Trechinotus*, his perileptines by *Perileptus*, and with *Paratrechus* and *Trechus* representing his trechines in the strict sense.

Trechini is a diverse group, with complex patterns of morphological variation, and has been subject to many different classification schemes. For example, some classifications view homaloderines as members of Trechina proper (e.g., Casale and Laneyrie 1982; Lorenz 2005). All classifications consider trechodines to be a distinct group, although the placement of *Perileptus* has varied. Jeannel (1926; 1927; 1928; 1930), Casale and Laneyrie (1982), and Lorenz (2005) treated perileptines as a group distinct from trechodines. Uéno (1989), however, considered *Perileptus* and related genera to be a trechodines, citing a misunderstanding by other authors of the structure of the male aedeagus in *Perileptus*. None of these classifications are based upon explict phylogenetic analyses, however.

In recent years larval structure and DNA sequences have been used in a few explicit phylogenetic studies within trechines. The most complete available larval data (Grebennikov 2008; Grebennikov and Maddison 2005), suggests that neither trechodines nor trechines are monophyletic, with trechodines forming a grade within Trechina. However, this result is not robust to the alternative assumptions employed by Grebennikov (2008). We have reanalyzed his data using his initial ordering assumptions, and find that relationships within trechines are more ambiguous than those shown in Grebennikov’s (2008) Fig. 3 (our Fig. 1B). Notably, some most-parsimonious trees have Trechodina and Trechina each monophyletic, and sister to each other (not shown). The only other paper to explicitly examine the phylogeny of trechines using modern analytical methods is that of Faille et al. (2010), which used 28S, 18S, and mitochondrial genes to infer the phylogeny of some European trechines, a very different question, with different taxon sampling, from the question of worldwide relationships examined here.

Our results (Figs 2–5, Table 4) confirm or refute several previous proposals. Trechina (including Jeannel’s homaloderines and the aepiine *Kenodactylus*) is monophyletic, supported by all three genes individually, and by analyses of the combined data. *Perileptus* is a trechodine, as supported by all three genes, and by the combined data, as predicted by Uéno (1989). There is weak evidence that trechodines are monophyletic, with *wingless* and the merged data in support, 28S ambiguous, and 18S speaking against monophyly.

### Relationships of Tasmanitachoides

In the original description of *Tasmanitachoides*, Erwin (1972) considered the genus to be “an early off-shoot of the tachyine lineage which gave rise to the Anillina”. He notes that they “show similarities to the trechines”, but view those similarities as symplesiomorphies. The genus placement in trechites has been examined in detail only once since then, in Grebennikov’s (2008) paper on larval characters. As Grebennikov reports, our DNA data indicates (as does his larval data) that *Tasmanitachoides* is not a tachyine, but instead shows affinity to trechines (Figs 2, 3, 5; Table 4). In our data, this relationship is supported by both ribosomal genes; we did not manage to acquire *wingless* sequence from *Tasmanitachoides*, and so that gene is at the moment mute on relationships of the genus.

### Monophyly of Zolini

Liebherr and Will’s (1999) study of female genitalic characters suggested that zolines were not monophyletic, with *Oopterus* and *Merizodus* appearing separately on their inferred phylogeny. However, as they note, the number of characters used was small enough to confer limited confidence in that result. Roig-Juñent and Chicchino’s (2001) study of adult structure, which included members of all subtribes of zolines, weakly supported monophyly of the group. 18S rDNA strongly supports monophyly of *Oopterus* + *Merizodus* + *Sloaneana* (Maddison, et al. 1999a; note that in that paper, *Oopterus helmsi* is referred to as “*Zolus helmsi*”).

Although our study cannot be a strong test of the monophyly of zolines, as we do not have members of two subtribes (Sinozolina and Chalteniina), the three genera we have examined (*Oopterus*, *Merizodus*, and *Sloaneana*) are strongly supported as a clade, in all three genes and in all analyses (Table 4).

### Monophyly of Pogonini

Pogonini is a tribe of about 70 species, most of which live in saline habitats (Bousquet and Laplante 1997). Except for the recently described genus, *Olegius* Komarov (1996), the monophyly of the tribe is not in question. Grebennikov and Bousquet (1999) found three synapomorphies in larvae that suggest monophyly of the tribe. Our results confirm this, with all genes and all analyses indicating that *Pogonus* + *Diplochaetus* + *Thalassotrechus* form a clade (Table 4).

### Monophyly of Bembidiini

As delimited in this paper, Bembidiini includes four subtribes: Bembidiina, a large group of over 1,200 species primarily found in temperate regions, and which includes the larger bembidiines; Tachyina, the second large group, mostly ground-dwelling, centered in warmer regions; Xystosomina, a primarily arboreal group most abundant in the Neotropics; and Anillina, containing very small, often blind, mostly soil-dwelling carabids. In some classifications, these groups are treated as separate tribes, in part as there is only one derived character that suggests that the group is monophyletic (the small terminal article of the maxillary and labial palps of adults, a character that occurs in some trechines, e.g., *Perileptus*), and in part as the subtribes are relatively uniform within themselves, but with several characters that distinguish them one from another. An analysis of larval characters (Grebennikov and Maddison 2005) gives no indication of monophyly of the tribe.

Our results are consistent with the view that Bembidiini is a heterogeneous group, with all three genes and the combined analysis indicating non-monophyly (Table 5), although with no consistent pattern of particular subgroups of Bembidiini being related to non-bembidiines. More details are provided under the discussions of each subtribe, below.

### Monophyly of Xystosomina

This subtribe was established by Erwin (1994) for six New World genera (four of which have arboreal members some species of which also use leaf-litter, and two having subcortical members) and one arboreal genus, *Philipis*, from tropical Australia. While no explicit phylogenetic analysis supporting monophyly of this group has been published, our data (which includes three of the more divergent genera) supports monophyly, with all three genes and the combined analyses indicating that xystosomines form a clade (Table 5).

### Monophyly of Tachyina

Monophyly of Tachyina exclusive of Xystosomina is supported by the obliquely notched front tibia of adults (Erwin 1994), a state apparently independently derived in anillines (as discussed below). This result is not consistent with larval data, which suggests that *Tachyta* (a tachyine) is more closely related to *Mioptachys* (a xystosomine) than it is to other tachyines (Grebennikov and Maddison 2005).

Bayesian analysis of 28S, *wingless*, and the merged matrix supports the monophyly of Tachyina (Table 5). However, this is result is not supported by Bayesian analysis of 18S rDNA, and parsimony analyses of all genes speak against monophyly of tachyines. A denser taxon sampling of both tachyines and xystosomines is needed to resolve this conflict.

### Monophyly and origin of Anillina

Most anillines are minute, blind, wingless, pale inhabitants of soil and deep leaf litter; members of a few genera have small eyes, e.g., *Nesamblyops* from New Zealand (Moore 1980) and *Microdipnodes* from Africa (Jeannel 1963). As most of the distinctive characteristics of anillines are expected to evolve in small beetles that live in soil, it is possible that anillines represent a grade that has repeatedly and independently evolved from above-ground trechites as those lineages went underground (Erwin 1982). Erwin proposed in particular that anillines may represent “a grade of numerous parallel lineages derived from *Paratachys* and allies.”

If Erwin (1982) is right that polyphyletic origin of anillines explains how these presumably slowly-dispersing beetles would be present on multiple continents and remote islands, then our sample of anillines from North America, Europe, and New Zealand should be a good test of his hypothesis. The only gene for which we have data from all four sampled genera is 28S. These data refute Erwin’s hypothesis, as the anillines are strongly supported as monophyletic (Fig. 2 and Table 4).

The exclusion of anillines from the Tachyina + Xystosomina clade (Fig. 7) suggests that the obliquely notched anterior tibia in anillines and Tachyina (Erwin 1982) arose independently in the two groups.

### Monophyly of Bembidiina

Bembidiina comprises all Bembidiini that do not belong to the other tribes; the group is defined by the lack of derived characters of its members. As such, evidence for monophyly is not evident in morphological data (Grebennikov 2008; Grebennikov and Maddison 2005). Our data show very limited evidence of monophyly of Bembidiina (only the Bayesian analysis of well-aligned nucleotides of *wingless* gives slight support) and evidence against monophyly from other analyses and from 28S rDNA. However, Bembidiina excluding *Phrypeus* is supported as monophyletic by 18S rDNA, *wingless*, and the merged matrix (Table 5). Further investigations of *Phrypeus* need to be conducted to see if it should be excluded from Bembidiina.

### Relationships within Bembidiina

The majority of species within subtribe Bembidiina belong to the genus *Bembidion*. *Bembidion* was regarded by Carl Lindroth (1963; 1980) and (as a result) most North American carabidologists as a very large genus comprising all non-tachyine, non-xystosomine, non-anilline bembidiines that possess a distinctive brush in the internal sac of the male genitalia, and with male foretarsomeres with adhesive setae arranged in a rows. Three groups of *Bembidiina* without a brush (*Phrypeus*, *Zecillenus*, *Bembidarenas* Erwin) have been excluded by Lindroth and from *Bembidion* on this basis, although the same was not done for some South American species also lacking a brush (e.g., members of the subgenus *Antiperyphanes*), which were still maintained within *Bembidion*. A similar brush is present in two groups outside *Bembidion* (in this classification): *Asaphidion* and some Xystosomina (Erwin 1994). The brush in xystosomines is likely convergent, as they share apomorphies that place them with a group that lacks a brush, the tachyines. *Asaphidion*, while having the brush, has distinctive dorsal texture and is unique within Trechitae of having adhesive setae on the foretarsomere of males arranged randomly, not in rows (Maddison, 1993). However, these traits are likely autapomorphies of *Asaphidion*, and thus not significant as evidence of relationships to other groups.

More recently, other groups have been excluded from *Bembidion* in most classifications, including *Ocys*, *Cillenus*, *Amerizus*, *Sinechostictus*, *Orzolina* Machado, and *Caecidium* Uéno. Thus, current classifications have a very large, poorly-defined genus *Bembidion* surrounded by a number of small “satellite” genera that are each defined in good part based upon autapomorphies. *Bembidion* in either the traditional or modern senses has no known synapomorphies of its members, and thus it would not be surprising if some of these satellite genera were found to be derived lineages within *Bembidion*. However, as there have been no comprehensive phylogenetic analyses at this level within Bembidiina, the lack of known derived states does not indicate that *Bembidion* as currently defined is non-monophyletic. Only some recent papers on larval characters (Grebennikov 2008; Grebennikov and Maddison 2005) employ cladistic analyses or numerical analyses of character matrices, and they do not have dense-enough taxon sampling to address most of these issues. Thus, there is little existing published evidence for or against the monophyly of *Bembidion* or other major subgroups within Bembidiina.

Our data suggest that some of these smaller genera are indeed outside of *Bembidion*. The distant relationship of *Phrypeus* to *Bembidion* is discussed above. Both *Amerizus* and *Ocys* fall outside of *Bembidion* in the broad sense in analyses of 28S rDNA, *wingless*, and the merged matrix (Figs 2, 4, 5). The placement of *Sinechostictus* has varied through time. These beetles very closely resemble in general habitus members of the *Ocydromus* complex of *Bembidion*, with which they have been placed by several authors (e.g., Antoine 1955; Jeannel 1941). In contrast, Perrault (1981) and Ortuño and Toribio (2005) considered *Sinechostictus* to be a group distinct from *Bembidion* based on spermathecal and aedeagal structures. Grebennikov (1997) found that *Sinechostictus* larvae lack some synapomorphies of *Bembidion* + *Asaphidion*, a result supported by further larval studies (Grebennikov 2008; Grebennikov and Maddison 2005). Our results corroborate this result, with *wingless* (Fig. 4) and the merged data (Fig. 5) suggesting that *Sinechostictus* falls outside of *Bembidion*.

On the other hand, some of the groups that have been recently considered outside of *Bembidion* are evidently derived members of that genus.

*Hydrium*, considered by most as a subgenus of *Bembidion* (e.g., Bousquet and Larochelle 1993; Lindroth 1963), has recently been removed from *Bembidion* (Lorenz 2005). Our results strongly indicate that *Bembidion (Hydrium) levigatum* is a *Bembidion*, and, among the taxa sampled, the sister group of *Bembidion (Metallina) dyschirinum*, with which it shares a number of characteristics, including an angulate shoulder margin.

*Cillenus* was considered by Lindroth (1980) and Toledano (2000) to be within *Bembidion*, but most current authors treat it as a separate genus (Lorenz 2005; Marggi et al. 2003; Ortuño and Toribio 2005; Perrault 1981). This separation from *Bembidion* is based in part on the unusual morphological traits of adult *Cillenus*, including a wide head and long mandibles. These features are likely derived features resulting from adaption to feeding on amphipods in their intertidal habitat (Green 1956; Lindroth 1980). Our results (Figs 2, 4, 5; Table 5) strongly support *Cillenus* as a member of *Bembidion*, near the *Ocydromus* complex.

When described, Lindroth (1980) separated *Zecillenus*, a lineage from New Zealand, from *Bembidion* because of the lack of a brush in the male aedeagus of *Zecillenus*. These beetles are rather distinct in general form, and have a number of unique characteristics, including unusual flanges on the elytra (Lindroth 1980). Our results indicate that they are highly derived *Bembidion*: among the taxa we sampled, they are strongly supported as belonging to *Bembidon* by all three genes and the merged matrix (Table 5), and are sister group to *Bembidion (Zeplataphus) tairuense* in 28S, *wingless*, and merged matrices. *Bembidion tairuense* is the only other *Bembidion* we have sampled from New Zealand, and we propose that *Zecillenus* is part of an endemic radiation of New Zealand *Bembidion*.

### Relationships of the major lineages of trechites

The only well-supported result we obtained about the relationships between the tribes or subtribes of trechites was the sister-group relationship between Tachyina and Xystosomina. This relationship is supported by the common presence of a recurrent groove on the elytra (Erwin 1994: 558).

## Conclusions

While our results have clarified the position of a number of enigmatic lineages within Trechitae, including *Tasmanitachoides*, *Cillenus*, and *Zecillenus*, our data have surprisingly little to say about deep relationships within Trechitae (Fig. 7). This is perhaps a result of the shortness of the deep branches in ribosomal gene trees (Figs 2, 3). Whether these might indicate a rapid radiation or limited ribosomal evolution during that period, they make inference of the relationships difficult.

Future work should increase taxon sampling, to split long branches in the tree, and add missing lineages. Additional genes, especially those with relatively longer lengths for the deeper branches (such as those seen in *wingless*, Fig. 4), are also needed. These efforts should increase our understanding of the phylogeny of this diverse group of small beetles.

## Appendix

**Table:** Locality data for specimens sequenced in this study. # is the D.R. Maddison voucher number.

**Taxon**	**#**	**Locality**
**Moriomorphini**		
*Sitaphe parallelipennis*	0669	Australia: North Queensland: Devil’s Thumb via Mossman
*Sitaphe parallelipenni*s	2247	Australia: North Queensland: Upper Whitehall Gully
*Tropopterus* sp.	2200	Chile: Reg. IX: Parque Nacional Nahuelbuta
**Psydrini**		
*Psydrus piceus*	1627	USA: New Mexico: Gila National Forest, Pine Flats Campground
*Nomius pygmaeus*	0893	USA: Arizona: Pima Co., Rincon Peak
**Trechini: Trechodina**		
*Cnides* sp.	1808, 0691	México: Sonora: Alamos, Rio Cuchujaqui
*Pachydesus* sp.	0678	Republic of South Africa: Kwazulu-Natal, Ngome Forest Reserve
*Perileptus areolatus*	1707	Spain: Madrid: Rio Cofio
*Perileptus areolatus*	0824	Russia: NW Caucasus: Krasnodar Dist., r. Belaya, Nikel
*Perileptus sloanei*	0767	Australia: Queensland: Gray’s Waterhole, Gayndah.
*Thalassophilus longicornis*	0823	Russia: NW Caucasus: Krasnodar Dist., r. Belaya, Nikel
*Trechodes bipartitus*	0705	Australia: Queensland: Cow Bay
*Trechodes jeanneli jeanneli*	0606	Madagascar: Fianarantsoa Province: Ranomafana National Park
*Trechosiella* sp.	1709	Republic of South Africa: North Cape Prov.: Farm Klipdam
*Tasmanitachoides fitzroyi*	1575, 0762	Australia: Queensland: Gayndah,Gray’s Waterhole
**Trechini: Trechina**		
*Trechus oregonensis*	0587	USA: Montana: Glacier Co., Divide Creek
*Omalodera limbata*	0571	Chile: Malleco Pr.: Coimallin area, 8.2 km NW Los Portones
*Homaloderodes germaini*	1066	Chile: Malleco Pr.: Coimallin area, 8.2 km NW Los Portones
*Kenodactylus audouini*	0670	Argentina: Tierra Del Fuego: 78 km E. of Ushuaia.
*Paratrechus* sp.	1076	Costa Rica: Cerro de la Muerte
*Trechinotus flavocinctus*	0575	Chile: Palena Pr.: Austral Highway km 67.9 (11 km S. Contao turnoff)
**Zolini**		
*Merizodus angusticollis*	0453	Chile: Valdivia Province: Rincón de la Piedra, 14.8 km SE Valdivia
*Oopterus* sp.	0387	New Zealand: South Island, Canterbury Prov, Arthur’s Pass Nat. Park
*Oopterus helmsi*	0354	New Zealand: South Island, Canterbury Prov, Arthur’s Pass Nat. Park
*Sloaneana tasmaniae*	0339	Australia: Tasmania: Mount Field N.P., Lake Dobson Rd., E end Wombat Moor
**Pogonini**		
*Pogonus (Pogonus) chalceus*	1711, 0679	Spain: Cádiz: El Puerto de Sta. Maria
*Thalassotrechus barbarae*	0703, 0530	USA: California: Marin Co., Tiburon Peninsula
**Bembidiini: Tachyina**		
*Lymnastis* sp.	0988	Malaysia: Sabah: Kinabatangan River
*Micratopus* sp.	0605	USA: Arizona: Pima Co., Tucson
*Paratachys vorax*	0410	USA: Arizona: Santa Cruz Co., Santa Cruz River near Tumacacori
*Elaphropus obesulus*	0411	USA: Arizona: Santa Cruz Co., Santa Cruz River near Tumacacori
*Elaphropus* cf. *nigrolimbatus*	0761	Republic of South Africa: Kwa-Zulu-Natal: Near Bayala
*Elaphropus* sp. 3	0713	Republic of South Africa: North Cape Prov.: Farm Klipdam
*Pericompsus laetulus*	0429	USA: Arizona: Pima Co., Arivaca Creek
*Polyderis rufotestacea*	0717, 0718	USA: Arizona: Gila Co., Gila River near Winkelman
*Tachys vittiger*	0760	USA: California: San Diego Co., Batiquitos Lagoon
*Tachys corax*	0604	USA: Arizona: Gila Co., Winkelman
*Tachyta nana inornata*	0573	USA: Mississippi: Noxubee Co., Noxubee Nat. Wildlife Refuge
**Bembidiini: Xystosomina**		
*Erwiniana hilaris*	0409	Ecuador: Sucumbios: Cuyabeno Faunal Reserve
*Erwiniana crassa*	0989	Ecuador: Orellana Province: Tiputini
*Mioptachys flavicauda*	0684	USA: Mississippi: Noxubee Co., Noxubee Nat. Wildlife Refuge
*Philipis bicolor*	0592	Australia: Queensland: Mt. Lewis Rd
**Bembidiini: Anillina**		
*Anillinus* (*langdoni* group) sp.	0690	USA: Georgia: Habersham Co., Big Panther Creek Trail
*Serranillus* sp.	1084	USA: North Carolina: Graham Co., Santeetlah Lake
*Typhlocharis armata*	0572, 1718	Spain: Cádiz: Tarifa
*Nesamblyops* sp.	0696	New Zealand: 3.5 km N Rapahoe
**Bembidiini: Bembidiina**		
*Asaphidion alaskanum*	0585	USA: Alaska: mile 412.3 Dalton Highway
*Asaphidion championi*	0574	Nepal: Prov. Karnali, Dolpo, Tripurakot Flußufer
*Asaphidion curtum*	0267	USA: Massachusetts: Norfolk Co., Jamaica Plain
*Amerizus* sp.	0576	USA: Utah: Abajo Mountains
*Ocys harpaloides*	0569	Belgium: Schorisse
*Phrypeus rickseckeri*	0776	USA: California: Del Norte Co., Smith River
*Phrypeus rickseckeri*	0692	USA: Montana: Jefferson Co., Boulder River at Galena Gulch
*Sinechostichus solarii*	0603	Italy: Tuscany: Vallombrosa
*Bembidion* (*Antiperyphanes*)sp. nr. *chilense*	0714	Peru: Pisac, tributary of Rio Urubamba, 3020m
*Bembidion* (*Hoquedela*) cf. *csikii*	0916	China: Yunnan Prov.: Gaoligong Shan, Nujiang Prefecture, 13.5 air km SSW of Gof Gonshan
*Bembidion (Cillenus) laterale*	0602	Germany: Wadden Sea, Mellum Island
*Bembidion (Notaphus) insulatum*	0444	USA: Arizona: Cochise Co., 2.2 km S of Willcox
*Bembidion (Metallina) dyschirinum*	0896	USA: Washington: Columbia Co., Blue Mountains
*Bembidion (Hydrium) levigatum*	0763	USA: Nebraska: Lancaster Co., Lincoln
*Bembidion (Ocydromus) mexicanum*	0260	USA: Arizona: Cochise Co., Turkey Creek, Chiricahua Mtns
*Bembidion (Phyla) obtusum*	0895	Canada: Ontario: Burlington
*Bembidion (Melomalus) planatum*	0601	Canada: British Columbia: Alexander Creek on highway 3
*Bembidion (Bembidion) quadrimaculatum dubitans*	0676	Canada: Alberta: Bayette Lake near Flatbush
*Bembidion (Zeplataphus) tairuense*	0607	New Zealand: Tekapo River Delta, Lake Benmore
*Bembidion* (*Zecillenus*)sp.	0595	New Zealand: Foxton Beach, Manuwatu
